# 7,8,9,10-Tetra­hydro-2-methyl­cyclo­hepta­[*b*]indol-6(5*H*)-one

**DOI:** 10.1107/S1600536808016498

**Published:** 2008-06-07

**Authors:** Makuteswaran Sridharan, Karnam J. Rajendra Prasad, Aimable Ngendahimana, Matthias Zeller

**Affiliations:** a Department of Chemistry, Bharathiar University, Coimbatore 641046, Tamil Nadu, India; b Department of Chemistry, Youngstown State University, One University Plaza, Youngstown, OH 44555, USA

## Abstract

The title compound, C_14_H_15_NO, was synthesized from 2-hydroxy­methyl­enecyclo­hepta­none *via* a Japp–Klingemann acid-catalyzed cyclization. The seven-membered ring exhibits a slightly distorted envelope conformation. N—H⋯O hydrogen bonds form a centrosymmetric dimer; C—H⋯O hydrogen bonds and π–π stacking inter­actions (the centers of the atoms involved in the stacking interaction are separated by 3.504 Å) give rise to another type of centrosymmetric dimer. In combination, these inter­actions create a stair-like chain of mol­ecules that inter­acts only loosely with neighboring chains *via* van der Waals inter­actions and weak C—H⋯π contacts.

## Related literature

For related literature on the synthesis, structure, anti­cancer and anti­depressant activities, and toxicity of functionalized cyclo­hept[*b*]indoles, see: Cornec *et al.* (1998 ▶); Joseph *et al.* (1999 ▶); Kinnick *et al.* (2006 ▶); Humphrey & Kuethe (2006 ▶, and references therein); Benoit *et al.* (2000 ▶); Kavitha & Rajendra Prasad (1999 ▶, and references therein). Brameld *et al.* (2008 ▶) describe small-mol­ecule conformational preferences derived from crystal structure data. Bernstein *et al.* (1995 ▶) present the use of the versatile graph-set analysis for the description of hydrogen bonds.
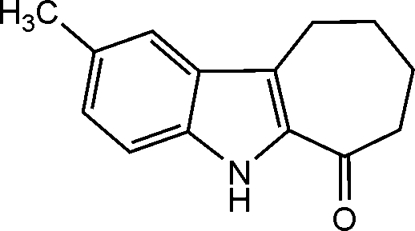


## Experimental

### 

#### Crystal data

C_14_H_15_NO
*M* *_r_* = 213.27
Monoclinic, 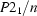

*a* = 11.461 (3) Å
*b* = 6.5062 (19) Å
*c* = 14.459 (4) Å
β = 92.310 (4)°
*V* = 1077.3 (6) Å^3^
*Z* = 4
Mo *K*α radiation
μ = 0.08 mm^−1^
*T* = 100 (2) K
0.48 × 0.10 × 0.08 mm

#### Data collection

Bruker SMART APEX CCD diffractometer
Absorption correction: multi-scan (*SADABS* as implemented in *APEX2*; Bruker, 2008 ▶) *T*_min_ = 0.731, *T*_max_ = 0.993
9984 measured reflections
2652 independent reflections
1581 reflections with *I* > 2σ(*I*)
*R*_int_ = 0.081

#### Refinement

*R*[*F* ^2^ > 2σ(*F* ^2^)] = 0.056
*wR*(*F* ^2^) = 0.135
*S* = 1.00
2652 reflections
146 parameters
H-atom parameters constrained
Δρ_max_ = 0.19 e Å^−3^
Δρ_min_ = −0.28 e Å^−3^

### 

Data collection: *APEX2* (Bruker, 2008 ▶); cell refinement: *APEX2*; data reduction: *APEX2*; program(s) used to solve structure: *SHELXTL* (Sheldrick, 2008 ▶); program(s) used to refine structure: *SHELXTL*; molecular graphics: *SHELXTL* and *Mercury* (Macrae *et al.*, 2006 ▶); software used to prepare material for publication: *SHELXTL* and *Mercury*.

## Supplementary Material

Crystal structure: contains datablocks global, I. DOI: 10.1107/S1600536808016498/fl2202sup1.cif

Structure factors: contains datablocks I. DOI: 10.1107/S1600536808016498/fl2202Isup2.hkl

Additional supplementary materials:  crystallographic information; 3D view; checkCIF report

## Figures and Tables

**Table 1:** Hydrogen-bond geometry (Å, °)

*D*—H⋯*A*	*D*—H	H⋯*A*	*D*⋯*A*	*D*—H⋯*A*
N1—H1⋯O1^i^	0.88	2.09	2.890 (2)	150
C4—H4a⋯O1^ii^	0.99	2.59	3.211 (3)	121
C2—H2*B*⋯*Cg*1^iii^	0.99	2.88	3.740 (2)	146
C5—H5b⋯C10^iv^	0.99	2.90	3.794 (2)	151

Symmetry codes: (i) 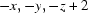; (ii) 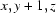; (iii) 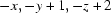; (iv) 
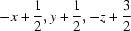
. *Cg*1 is the centroid of the C8–C13 ring.

**Fig. 1.**
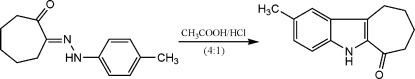
Synthesis of the title compound

**Fig. 2.**
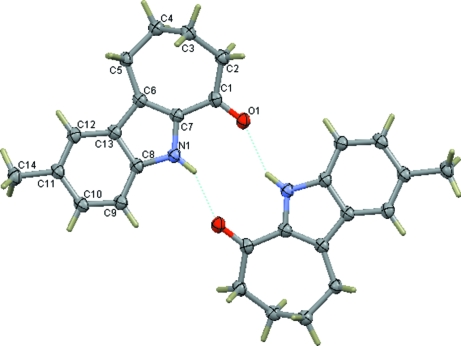
Thermal ellipsoid plot of one of the N—H···O bonded dimers with the atom labeling scheme. Displacement ellipsoids are shown at the 50% probability level, hydrogen atoms are shown as capped sticks.

**Fig. 3.**
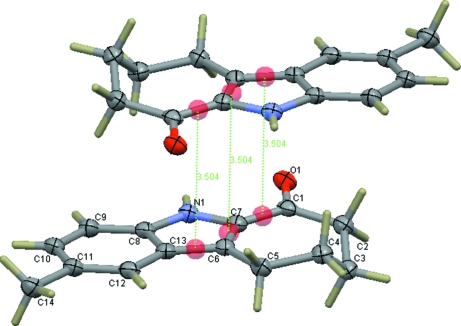
Thermal ellipsoid plot of one of the π-stacked dimers. Red spheres are centers of π bonds involved in the π-stacking interactions, green dotted lines represent π···π contacts. Atom and bond styles as in Figure 2.

**Fig. 4.**
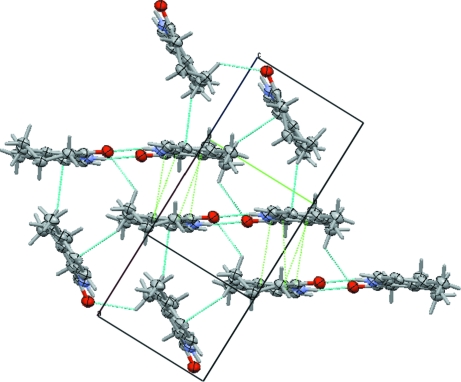
Packing view of the title crystal. Blue dotted lines represent N—H···O, C—H···O and C—H···π interactions. Green dotted lines are π···π contacts. Atom and bond styles as in Figure 2.

## supplementary crystallographic information

### Comment

Development of new methods towards the synthesis of functionalized cyclohept[*b*]indoles is currently attractive to organic chemists due to the discovery of anti-cancer and anti-depressant activities associated with this system (Cornec *et al.*, 1998; Joseph *et al.* 1999). Synthetic studies on the large family of indoles have been documented extensively because of their structural diversity and association with their wide spectrum of pharmacological potential. Synthetic approaches to prepare cyclohept[*b*]indoles have been described in the literature and examined for central nervous system activity and reported to be active as antidepressants (Kinnick *et al.*, 2006; Humphrey & Kuethe, 2006, and references therein). The design of molecules that spontaneously organize into a helical architecture is of considerable interest because of their fascinating structural features as well as their potential applications. Benoit *et al.* (2000) have reported the toxicity and structure activity relationship of benzocyclohept[*b*]indoles and these were found to be potent anti-inflammatory and anti-cancer agents. Therefore, much effort has been directed towards the development of efficient methodologies for the construction of heterocyclo-fused cyclohept[*b*]indoles. Based on the interesting features of these compounds we reported the synthesis and utility of cyclohept[*b*]indoles and their substituted analogs (Kavitha & Rajendra Prasad 1999, and references therein).

Cyclization of 2-hydroxymethylenecycloheptanone under Japp-Klingemann conditions using acetic acid and HCl in a 4:1 ratio (Kent's reagent) as the catalyst furnished the title compound (Fig. 1). Details of the synthesis of the title compound were reported by Kavitha & Rajendra Prasad (1999). An *ORTEP* style representation of the title compound is given in Fig 2.

The *sp*^2^ hybridized section of the molecule is essentially planar with an r.m.s. deviation from the mean plane of only 0.052 Å. Of the methylene carbon atoms C3 is deviates the most (0.786 (3) Å ) from this plane. Deviations for C2 and C4 are 0.252 (2) and -0.157 (3) Å, respectively. The seven membered ring thus is best described as having a slightly distorted envelope conformation (Brameld *et al.* 2008). All bond distances and angles in the structure of the title compound are in the expected ranges.

Via a pair of N—H···O hydrogen bonds the molecules form centrosymmetric dimers with an *R*^2^_2_(10) graph set motif (Bernstein *et al.*, 1995) in the solid state (Table 1, Fig. 3). The keto oxygen atom also acts as acceptor for a weaker C—H···O hydrogen bond from another neighboring molecule. The same neighboring molecule also acts as a partner for a π-π stacking interaction across an inversion center. The π moieties of these thus formed π-stacked dimers are slipped against each other and only the atoms C1, C6, C7 and C13 and their inversion symmetry related counterparts in the neighboring molecule are involved in the interaction of the π systems with a distance of about 3.5 Å between the planes (Fig. 3). Distances between the slipped centroids of the pyrrole rings are given in table 1. The main planes of the molecules are connected *via* the N—H···O and C—H···O hydrogen bonds and the π-π stacking interactions are all aligned roughly in parallel to each other. The two types of dimers created by the N—H···O and  C—H···O interactions do each share one molecule, which extends the molecules held together *via* these strong to medium interactions into a flight of stairs like chain of molecules. Neighboring chains are only loosly connected *via* van der Waals interactions and weak C—H···π contacts (Table 1, Fig. 4).

### Experimental

A solution of 2-(2-(4-methylphenyl)hydrazono)cycloheptanone (0.230 g, 0.001 mol) in a mixture of acetic acid (20 ml) and concentrated hydrochloric acid (5 ml) was heated to reflux on an oil bath pre-heated to 398–403 K for 2 h. The reaction was monitored by TLC. After completion of the reaction the contents were cooled and poured into icewater with stirring. The separated brown solid was filtered and purified by passing through a column of silica gel and eluting with a petroleum ether-ethyl acetate mixture (95:5) to yieldthe title compound (171 mg, 80%). The product thus obtained was recrystallized using ethanol, m.p. 451–453 K.

### Refinement

All hydrogen atoms were added in calculated positions with a C—H bond distances of 0.99 (methylene), 0.95 (aromatic) and 0.98 Å (methyl) and an N—H distance of 0.88 Å. They were refined with isotropic displacement parameteres *U*_iso_ of 1.5 (methyl) or 1.2 times *U*_eq_ (all others) of the adjacent carbon or nitrogen atom. The s.u. values of the cell parameters are taken from the software recognizing that the values are unreasonably small.

### Crystal data

**Table:**

C_14_H_15_NO	*F*(000) = 456
*M**_r_* = 213.27	*D*_x_ = 1.315 Mg m^−^^3^
Monoclinic, *P*2_1_/*n*	Mo *K*α radiation, λ = 0.71073 Å
*a* = 11.461 (3) Å	Cell parameters from 1265 reflections
*b* = 6.5062 (19) Å	θ = 2.3–24.5°
*c* = 14.459 (4) Å	µ = 0.08 mm^−^^1^
β = 92.310 (4)°	*T* = 100 K
*V* = 1077.3 (6) Å^3^	Plate, colourless
*Z* = 4	0.48 × 0.10 × 0.08 mm

### Data collection

**Table:**

Bruker SMART APEX CCD diffractometer	2652 independent reflections
Radiation source: fine-focus sealed tube	1581 reflections with *I* > 2σ(*I*)
graphite	*R*_int_ = 0.081
ω scans	θ_max_ = 28.3°, θ_min_ = 2.2°
Absorption correction: multi-scan (*SADABS* as implemented in *APEX2*; Bruker, 2008)	*h* = −15→15
*T*_min_ = 0.731, *T*_max_ = 0.993	*k* = −8→8
9984 measured reflections	*l* = −19→19

### Refinement

**Table:**

Refinement on *F*^2^	Primary atom site location: structure-invariant direct methods
Least-squares matrix: full	Secondary atom site location: difference Fourier map
*R*[*F*^2^ > 2σ(*F*^2^)] = 0.056	Hydrogen site location: inferred from neighbouring sites
*wR*(*F*^2^) = 0.135	H-atom parameters constrained
*S* = 1.00	*w* = 1/[σ^2^(*F*_o_^2^) + (0.0596*P*)^2^] where *P* = (*F*_o_^2^ + 2*F*_c_^2^)/3
2652 reflections	(Δ/σ)_max_ < 0.001
146 parameters	Δρ_max_ = 0.19 e Å^−^^3^
0 restraints	Δρ_min_ = −0.28 e Å^−^^3^

### Special details

**Table:**

Geometry. All e.s.d.'s (except the e.s.d. in the dihedral angle between two l.s. planes) are estimated using the full covariance matrix. The cell e.s.d.'s are taken into account individually in the estimation of e.s.d.'s in distances, angles and torsion angles; correlations between e.s.d.'s in cell parameters are only used when they are defined by crystal symmetry. An approximate (isotropic) treatment of cell e.s.d.'s is used for estimating e.s.d.'s involving l.s. planes.
Refinement. Refinement of *F*^2^ against ALL reflections. The weighted *R*-factor *wR* and goodness of fit *S* are based on *F*^2^, conventional *R*-factors *R* are based on *F*, with *F* set to zero for negative *F*^2^. The threshold expression of *F*^2^ > σ(*F*^2^) is used only for calculating *R*-factors(gt) *etc*. and is not relevant to the choice of reflections for refinement. *R*-factors based on *F*^2^ are statistically about twice as large as those based on *F*, and *R*- factors based on ALL data will be even larger.

### Fractional atomic coordinates and isotropic or equivalent isotropic displacement parameters (Å^2^)

**Table:**

	*x*	*y*	*z*	*U*_iso_*/*U*_eq_	
C1	0.13517 (16)	0.2801 (3)	1.08139 (13)	0.0251 (4)	
C2	0.21105 (17)	0.3796 (3)	1.15581 (13)	0.0284 (5)	
H2A	0.2532	0.2688	1.1901	0.034*	
H2B	0.1589	0.4463	1.1998	0.034*	
C3	0.30146 (17)	0.5391 (3)	1.12828 (14)	0.0283 (5)	
H3A	0.3491	0.5786	1.1841	0.034*	
H3B	0.3544	0.4750	1.0841	0.034*	
C4	0.24910 (17)	0.7329 (3)	1.08433 (13)	0.0280 (5)	
H4A	0.1735	0.7618	1.1125	0.034*	
H4B	0.3018	0.8500	1.0988	0.034*	
C5	0.22933 (17)	0.7184 (3)	0.97993 (13)	0.0272 (5)	
H5A	0.1874	0.8438	0.9586	0.033*	
H5B	0.3066	0.7194	0.9516	0.033*	
C6	0.16294 (15)	0.5361 (3)	0.94335 (13)	0.0241 (4)	
C7	0.12670 (16)	0.3572 (3)	0.98660 (13)	0.0243 (4)	
C8	0.06994 (16)	0.3237 (3)	0.83704 (13)	0.0244 (4)	
C9	0.02351 (16)	0.2566 (3)	0.75210 (13)	0.0274 (5)	
H9	−0.0126	0.1257	0.7455	0.033*	
C10	0.03204 (16)	0.3877 (3)	0.67779 (14)	0.0287 (5)	
H10	−0.0003	0.3463	0.6192	0.034*	
C11	0.08746 (17)	0.5823 (3)	0.68576 (13)	0.0275 (5)	
C12	0.13502 (16)	0.6455 (3)	0.77013 (14)	0.0271 (5)	
H12	0.1734	0.7746	0.7759	0.032*	
C13	0.12601 (16)	0.5160 (3)	0.84787 (13)	0.0245 (4)	
C14	0.09470 (18)	0.7160 (3)	0.60049 (14)	0.0339 (5)	
H14A	0.1211	0.8539	0.6187	0.051*	
H14B	0.0175	0.7250	0.5691	0.051*	
H14C	0.1502	0.6555	0.5584	0.051*	
N1	0.07063 (13)	0.2311 (2)	0.92167 (10)	0.0253 (4)	
H1	0.0402	0.1101	0.9332	0.030*	
O1	0.07986 (11)	0.1233 (2)	1.10088 (9)	0.0300 (4)	

### Atomic displacement parameters (Å^2^)

**Table:**

	*U*^11^	*U*^22^	*U*^33^	*U*^12^	*U*^13^	*U*^23^
C1	0.0207 (10)	0.0302 (10)	0.0251 (11)	0.0036 (8)	0.0076 (8)	0.0000 (8)
C2	0.0258 (10)	0.0348 (11)	0.0248 (11)	0.0005 (9)	0.0034 (9)	0.0010 (9)
C3	0.0240 (10)	0.0350 (11)	0.0258 (11)	0.0028 (9)	0.0002 (8)	−0.0005 (9)
C4	0.0233 (10)	0.0321 (11)	0.0286 (11)	0.0013 (9)	0.0017 (9)	−0.0010 (9)
C5	0.0260 (10)	0.0312 (11)	0.0246 (11)	−0.0014 (9)	0.0036 (9)	0.0008 (9)
C6	0.0183 (9)	0.0305 (10)	0.0237 (10)	0.0030 (8)	0.0032 (8)	−0.0015 (8)
C7	0.0196 (10)	0.0294 (10)	0.0241 (11)	0.0012 (8)	0.0037 (8)	−0.0021 (8)
C8	0.0182 (10)	0.0309 (10)	0.0245 (11)	0.0007 (8)	0.0050 (8)	0.0007 (9)
C9	0.0228 (10)	0.0337 (11)	0.0260 (11)	−0.0034 (9)	0.0049 (8)	−0.0016 (9)
C10	0.0206 (10)	0.0429 (12)	0.0230 (11)	−0.0005 (9)	0.0051 (8)	−0.0025 (9)
C11	0.0210 (10)	0.0380 (12)	0.0239 (11)	−0.0015 (9)	0.0048 (8)	0.0009 (9)
C12	0.0204 (10)	0.0327 (11)	0.0284 (11)	0.0000 (9)	0.0060 (8)	0.0019 (9)
C13	0.0170 (9)	0.0321 (11)	0.0246 (11)	0.0008 (8)	0.0044 (8)	−0.0013 (9)
C14	0.0297 (12)	0.0439 (13)	0.0284 (12)	−0.0028 (10)	0.0067 (9)	0.0052 (10)
N1	0.0226 (9)	0.0292 (9)	0.0243 (9)	−0.0015 (7)	0.0035 (7)	0.0008 (7)
O1	0.0311 (8)	0.0309 (8)	0.0283 (8)	−0.0012 (6)	0.0070 (6)	0.0028 (6)

### Geometric parameters (Å, °)

**Table:**

C1—O1	1.239 (2)	C7—N1	1.385 (2)
C1—C7	1.459 (3)	C8—N1	1.364 (2)
C1—C2	1.502 (3)	C8—C9	1.389 (3)
C2—C3	1.531 (3)	C8—C13	1.412 (3)
C2—H2A	0.9900	C9—C10	1.378 (3)
C2—H2B	0.9900	C9—H9	0.9500
C3—C4	1.524 (3)	C10—C11	1.420 (3)
C3—H3A	0.9900	C10—H10	0.9500
C3—H3B	0.9900	C11—C12	1.379 (3)
C4—C5	1.520 (3)	C11—C14	1.514 (3)
C4—H4A	0.9900	C12—C13	1.412 (3)
C4—H4B	0.9900	C12—H12	0.9500
C5—C6	1.494 (3)	C14—H14A	0.9800
C5—H5A	0.9900	C14—H14B	0.9800
C5—H5B	0.9900	C14—H14C	0.9800
C6—C7	1.393 (3)	N1—H1	0.8800
C6—C13	1.434 (3)		
			
O1—C1—C7	118.77 (18)	N1—C7—C6	109.23 (17)
O1—C1—C2	118.56 (17)	N1—C7—C1	116.39 (17)
C7—C1—C2	122.64 (18)	C6—C7—C1	134.38 (18)
C1—C2—C3	118.96 (16)	N1—C8—C9	130.00 (18)
C1—C2—H2A	107.6	N1—C8—C13	107.80 (17)
C3—C2—H2A	107.6	C9—C8—C13	122.19 (18)
C1—C2—H2B	107.6	C10—C9—C8	117.27 (19)
C3—C2—H2B	107.6	C10—C9—H9	121.4
H2A—C2—H2B	107.0	C8—C9—H9	121.4
C4—C3—C2	114.21 (16)	C9—C10—C11	122.29 (19)
C4—C3—H3A	108.7	C9—C10—H10	118.9
C2—C3—H3A	108.7	C11—C10—H10	118.9
C4—C3—H3B	108.7	C12—C11—C10	119.84 (18)
C2—C3—H3B	108.7	C12—C11—C14	121.11 (18)
H3A—C3—H3B	107.6	C10—C11—C14	119.04 (18)
C5—C4—C3	113.79 (16)	C11—C12—C13	119.17 (18)
C5—C4—H4A	108.8	C11—C12—H12	120.4
C3—C4—H4A	108.8	C13—C12—H12	120.4
C5—C4—H4B	108.8	C12—C13—C8	119.21 (18)
C3—C4—H4B	108.8	C12—C13—C6	133.15 (18)
H4A—C4—H4B	107.7	C8—C13—C6	107.63 (16)
C6—C5—C4	116.99 (16)	C11—C14—H14A	109.5
C6—C5—H5A	108.1	C11—C14—H14B	109.5
C4—C5—H5A	108.1	H14A—C14—H14B	109.5
C6—C5—H5B	108.1	C11—C14—H14C	109.5
C4—C5—H5B	108.1	H14A—C14—H14C	109.5
H5A—C5—H5B	107.3	H14B—C14—H14C	109.5
C7—C6—C13	105.92 (16)	C8—N1—C7	109.40 (16)
C7—C6—C5	131.40 (17)	C8—N1—H1	125.3
C13—C6—C5	122.66 (17)	C7—N1—H1	125.3
			
O1—C1—C2—C3	165.37 (17)	C9—C10—C11—C12	0.0 (3)
C7—C1—C2—C3	−12.8 (3)	C9—C10—C11—C14	179.14 (17)
C1—C2—C3—C4	64.2 (2)	C10—C11—C12—C13	−1.0 (3)
C2—C3—C4—C5	−88.3 (2)	C14—C11—C12—C13	179.88 (17)
C3—C4—C5—C6	51.7 (2)	C11—C12—C13—C8	0.9 (3)
C4—C5—C6—C7	−9.5 (3)	C11—C12—C13—C6	−178.67 (19)
C4—C5—C6—C13	172.25 (17)	N1—C8—C13—C12	−178.80 (16)
C13—C6—C7—N1	0.32 (19)	C9—C8—C13—C12	0.3 (3)
C5—C6—C7—N1	−178.10 (18)	N1—C8—C13—C6	0.8 (2)
C13—C6—C7—C1	−179.5 (2)	C9—C8—C13—C6	179.93 (16)
C5—C6—C7—C1	2.0 (3)	C7—C6—C13—C12	178.9 (2)
O1—C1—C7—N1	−9.3 (3)	C5—C6—C13—C12	−2.5 (3)
C2—C1—C7—N1	168.83 (16)	C7—C6—C13—C8	−0.71 (19)
O1—C1—C7—C6	170.51 (19)	C5—C6—C13—C8	177.89 (16)
C2—C1—C7—C6	−11.3 (3)	C9—C8—N1—C7	−179.65 (18)
N1—C8—C9—C10	177.61 (18)	C13—C8—N1—C7	−0.7 (2)
C13—C8—C9—C10	−1.3 (3)	C6—C7—N1—C8	0.2 (2)
C8—C9—C10—C11	1.1 (3)	C1—C7—N1—C8	−179.91 (15)

### Hydrogen-bond geometry (Å, °)

**Table:**

*D*—H···*A*	*D*—H	H···*A*	*D*···*A*	*D*—H···*A*
N1—H1···O1^i^	0.88	2.09	2.890 (2)	150.
C4—H4a···O1^ii^	0.99	2.59	3.211 (3)	121
C2—H2B···Cg1^iii^	0.99	2.88	3.740 (2)	146
C5—H5b···C10^iv^	0.99	2.90	3.794 (2)	151

Symmetry codes: (i) −*x*, −*y*, −*z*+2; (ii) *x*, *y*+1, *z*; (iii) −*x*, −*y*+1, −*z*+2; (iv) −*x*+1/2, *y*+1/2, −*z*+3/2.
